# COVID-19 Vaccination and Cancer, the Need for more Data

**DOI:** 10.31557/APJCP.2021.22.10.3053

**Published:** 2021-10

**Authors:** Seyed Alireza Javadinia, James S Welsh, Seyed Alireza Mosavi Jarrahi

**Affiliations:** 1 *Clinical Research Development Unit, Hospital Research Development Committee, Sabzevar University of Medical Sciences, Sabzevar. *; 2 *Cancer Research Center, Shahid Beheshti University of Medical Sciences, Tehran, Iran. *; 3 *Edward Hines Jr VA Hospital and Loyola University Chicago Stritch School of Medicine. Chicago, IL, USA. *

**Dear Editor,**

The ongoing coronavirus disease (COVID-19) pandemic imposes severe health effects all around the world (Basu, 2020; Dewi et al., 2020). Infected patients who suffer from comorbidities such as underlying malignancy might be at higher risks for intensive care unit admission, mortality, and morbidity (Shahidsales et al., 2021; Taghizadeh-Hesary et al., 2021). On the other hand, due to the lack of resources or even “coronaphobia”, the diagnosis and oncologic treatment of patients might be delayed resulting in the presenting with more advanced disease (Kregting et al., 2021; Soroosh et al., 2020; Yadav et al., 2020; Chakraborty et al., 2020).

Currently, an optimal medical treatment of COVID-19 is lacking, therefore, suffering from malignancies should be a high priority for vaccination programs as the best preventive approach (Corti et al., 2021). Various types of COVID19 vaccines including inactivated virus, protein subunits, replication incompetent adenoviral vector, DNA-based, and mRNA-based ones have been developed (Hwang et al., 2021). However, cancer patients have been excluded from the most of the initial trials assessing the efficacy and safety of these vaccines (Corti et al., 2021). 

Serval cohort studies have assessed the safety and seroconversion after COVID-19 vaccination in cancer patients. These studies enrolled patients with various malignancies and evaluated the efficacy based on measuring serum levels of anti-spike and neutralizing Ig G antibodies. The type of vaccines in all of these studies were COVID-19 mRNA vaccines (Addeo et al., 2021; Ariamanesh et al., 2021) except a preprint by Ariamanesh et al., (2021) which used inactivated SARS-CoV-2 vaccine (BBIBP-CorV). 

These studies have shown that COVID-19 vaccination is a safe procedure in cancer patients and can induce seroconversion in considerable portion of patients after two doses of vaccination. However, the rate of seropositivity was considerably lower in those suffering from hematologic malignancies and patients actively receiving chemotherapy (Eliakim-Raz et al., 2021; Heudel et al., 2021; Goshen-Lago et al., 2021; Massarweh et al., 2021). 

Therefore, we recommend all patients suffering from cancer should receive full doses of vaccinations. A third boost dose may be considered in both patients undergoing active chemotherapeutic treatment and those suffering from hematologic malignancies. Finally, we feel that cancer patients should be allowed to and encouraged to enroll in clinical trials to assess the effects of different cancer types and various treatment regimens including chemotherapy, targeted therapy, immunotherapy and radiotherapy.

While the benefit of vaccination outweighs all the risks (direct acquiring the infection) and indirect (treatment, diagnosis, and system delays) imposed on cancer patients, the following gaps in understanding COVID 19 vaccinations in cancer patients need to be addressed:

1) Lack of vaccine efficacy data in cancer patients

2) The durability vaccine protection compares to general population

3) The immune response (humoral and cellular) among cancer patients

4) Cancer treatment modalities and their interaction with vaccine immunogenicity.

## Custom

Study concept and design: S. A. J.; drafting of the manuscript: J.S.W.; critical revision of the manuscript for important intellectual content: S.A.M.J.
